# Winter Bottom Beehive Cadavers as a Tool for Assessing *Nosema ceranae* Infestation Intensity in Honeybee Colonies in Regions with Different Beekeeping Densities in Slovakia

**DOI:** 10.3390/microorganisms14030694

**Published:** 2026-03-19

**Authors:** Simona Hriciková, Martin Staroň, Lucia Sabová, Monika Sučik

**Affiliations:** 1 Department of Biology and Physiology, University of Veterinary Medicine and Pharmacy in Košice, Komenského 73, 041 81 Košice, Slovakia; 2 National Agricultural and Food Centre, Institute of Apiculture Liptovský Hrádok, Gašperíkova 599, 033 80 Liptovský Hrádok, Slovakia; martin.staron@nppc.sk; 3 Department of Pharmacology and Toxicology, University of Veterinary Medicine and Pharmacy in Košice, Komenského 73, 041 81 Košice, Slovakia; lucia.sabova@uvlf.sk

**Keywords:** *Nosema apis*, *Nosema ceranae*, nosemosis, honeybees

## Abstract

Honeybee (*Apis mellifera*) colony density is frequently assumed to influence the level of *Nosema ceranae* infestation in managed colonies. In Slovakia, winter bottom beehive debris (dead worker bees) is routinely collected between January and February, providing a unique and uniform material for evaluating the degree of *Nosema* infestation prior to the breeding season. This study assesses the suitability of winter hive debris for estimating the infestation intensity of *Nosema* species and examines whether regional differences in beekeeping density are associated with variation in *Nosema ceranae* infestation levels. A total of 6221 samples from 43 Slovak districts collected between 2022 and 2024 were examined using microscopy confirmed by duplex PCR. *Nosema ceranae* was detected in 74.3% of samples, while *Nosema apis* was not detected. Although higher colony densities tended to be associated with increased proportions of moderately and strongly infested colonies, statistical modelling confirmed a statistically significant but modest positive association between colony density and infestation intensity. These results indicate that winter bottom beehive debris is a valuable material for assessing *Nosema* infestation pressure at the colony and regional levels, while also highlighting the contribution of additional environmental and management factors.

## 1. Introduction

Honeybees (*Apis mellifera*) play a crucial role in global ecosystems and agriculture as primary pollinators of wild plants and economically important crops, contributing significantly to biodiversity and food security [1]. However, honeybee populations worldwide are under increasing threat due to multiple environmental pressures, including habitat loss, pesticide exposure, climate changes, and pathogen infestations [2]. Among the most widespread and damaging pathogens affecting honeybee health is *Nosema* spp., a microsporidian parasite responsible for nosemosis, a disease that impairs colony productivity and survival [3,4].

Two primary species of *Nosema* are known to infect bees, *Nosema apis* and *Nosema ceranae*. While *N. apis* is commonly associated with infections in cooler climates, *N. ceranae*, an emerging and globally prevalent species is often considered more virulent [5,6]. These pathogens infect the midgut epithelial cells of bees, resulting in disrupted digestion, malnutrition, reduced foraging abilities, and shortened lifespans. In severe outbreaks, *Nosema* infections can compromise the immune systems of entire colonies, exacerbate exposure to secondary stressors, and contribute to colony collapse disorder (CCD) [7].

*N. apis* has been recognized as a honeybee pathogen since the early 20th century. It primarily infects the midgut of adult bees, with symptoms often manifesting as dysentery, reduced foraging ability, and increased mortality, particularly in the spring when the parasite population is the highest. Historically, the control methods for *N. apis* included the use of antibiotics like fumagillin; however, resistance and regulatory issues have complicated these strategies [3,6].

*N. ceranae*, initially discovered in Asian honeybees (*Apis cerana*), has emerged as a significant threat to *A. mellifera* since its introduction to Europe and North America in the early 2000s [5]. Research has shown that *N. ceranae* can be more virulent than *N. apis*, often leading to severe colony losses [8,9]. It is characterized by its ability to replicate in high temperatures, which can exacerbate the impact on colonies during periods of stress, such as during drought or heat waves [8,10].

The higher prevalence of *N. ceranae* compared to *N. apis* in the observed colonies is related to its ability to better adapt to higher temperatures and stressful conditions, which may lead to a more significant negative impact on colony vitality. Therefore, it is assumed that colonies infected with *N. ceranae* will exhibit a higher mortality rate and reduced productivity compared to colonies infected with *N. apis*, especially during periods of climatic stress [11].

Both species of *Nosema* can cause sub-lethal effects on honeybee behaviour and physiology, impacting crucial aspects such as foraging efficiency, reproductive success, and overall colony vitality [12,13]. The infection can also compromise the immune system of bees, making them more susceptible to other diseases and environmental stressors. Studies have linked high *Nosema* loads with increased levels of stress hormones in bees, further influencing their health and behaviour [11,14]. The cumulative findings emphasize the importance of monitoring *Nosema* infections to mitigate their effects on honeybee health and ensure sustainable apiculture [15,16,17].

Although *Nosema ceranae* has traditionally been classified within the genus *Nosema*, recent taxonomic revisions based on molecular phylogenetics have proposed its reclassification into the genus *Vairimorpha*. However, this reclassification remains a subject of ongoing scientific debate, and both nomenclatures are still used in the current literature. In this study, we follow the conventional nomenclature for clarity and comparability with previous research.

The decline of honeybee populations due to *Nosema* infection poses a significant threat not only to beekeepers but also to agricultural ecosystems reliant on pollination. With honeybees responsible for a substantial portion of the global food supply, understanding and mitigating the impact of *Nosema* is crucial for maintaining healthy bee populations and ensuring agricultural productivity [18,19].

Accurate monitoring of *Nosema* infections are pivotal for understanding their impact and managing their spread [20]. Over recent decades, there has been a concerning rise in honeybee colony losses, significantly influenced by microsporidians such as *Nosema* spp. [21,22,23]. As it is important to know the epidemiological situation of infection of individual diseases, and therefore it is necessary to carry out screening observations in a given area, to take measures and improve the health situation of bees in Slovakia.

Detection of *Nosema* spp. in honeybee colonies traditionally relies on a combination of microscopic and molecular methods, which together provide a comprehensive assessment of the colony’s infection status. Light microscopy remains one of the most widely used diagnostic techniques in routine epidemiological screening. It enables rapid and reasonably accurate determination of infection intensity based on the number of spores in homogenised abdominal tissues of worker bees and allows subsequent categorisation of infection (weak, moderate, strong), which is practical for evaluating infection pressure at both colony and regional levels [3,10]. Its principal advantages include speed, low cost, and the ability to process large sample volumes, although it is limited by the inability to reliably distinguish between *N. apis* and *N. ceranae*, which are morphologically very similar [9,24].

For this reason, microscopy is commonly supplemented with molecular methods, particularly species-specific PCR, which ensures unequivocal identification of the pathogen based on its genetic signature and enables detection of low-level infections that may be microscopically unrecognisable [25]. In large-scale screening settings, duplex PCR for simultaneous detection of both species in a single reaction has proven practical; published and validated protocols show adequate sensitivity for epidemiological use and strong concordance with microscopic examination in samples with moderate and high spore loads [26]. Combining microscopy with PCR thus merges rapid semi-quantification with precise species identification and represents a robust methodological framework for studies monitoring prevalence, regional trends, and temporal changes in infection intensity [3,18].

From previous research on the prevalence of *Nosema* spp. in Slovakia [10], it can be stated with certainty that *Nosema* infection is widespread in honeybees. We therefore expect that this research will supplement the information on the general distribution of this parasite and confirm the dominance of the species *N. ceranae* over *N. apis*. We assume that the species structure will be consistent with previous studies, with possible regional differences in the intensity of infection. Seasonal and climatic factors may also influence the prevalence, but we do not think that they will change the dominance of *N. ceranae*.

Based on the above, the primary aim of this study was to evaluate winter bottom beehive debris as a consistently and practical material for assessing the intensity of *Nosema* infestation in honeybee colonies across Slovakia. A secondary objective was to determine whether differences in regional beekeeping density are associated with variation in *Nosema ceranae* infestation levels. Specifically, we tested the hypotheses that (i) *Nosema ceranae* is the predominant *Nosema* species detected in winter debris samples; (ii) colonies originating from districts with higher beekeeping density exhibit higher infestation intensity; and (iii) regional differences in infestation intensity cannot be explained by colony density alone despite its statistically significant contribution, implying the involvement of additional factors.

## 2. Materials and Methods

### 2.1. Sample Collection

For screening observation, 6221 samples of dead worker bees were collected during years 2022, 2023 and 2024 from 43 districts of Slovakia. Samples from apiaries were collected annually, except for apiaries with a smaller number of colonies from hobby beekeepers who do not have their colonies examined annually. The colonies were selected based on random sampling. Bees were collected from the hive bottom during January and February, representing the natural mortality of bees during the wintering period.

Samples were stored in clean, labelled breathable paper bags. Collected bees were preserved at −20 °C until laboratory analysis to minimize degradation.

### 2.2. Preparation of Samples for Microscopic Analysis

Bee abdomens, in number 50 from each bee colony, were extracted and transferred to a mortar. Each sample was homogenized precisely in 15 mL of distilled water using a sterile porcelain pestle. The homogenate was filtered through a gauze to remove debris and large tissue fragments.

### 2.3. Microscopic Techniques

A drop of the filtered homogenate was placed onto a glass slide and covered with a coverslip. No staining was performed for standard light microscopy observations. Observations were conducted using a digital microscope VisiScope^®^ BL254 T1 (VWR, Avantor, Radnor, PA, USA) equipped with a 40× objective lens (total magnification: 400×) using phase contrast. *Nosema* spores were identified based on morphological criteria such as specific size 4–6 × 2–4 µm (*N. apis*) and 3.3–5.5 × 2.3–3.0 µm (*N. ceranae*) and specific oval shape.

### 2.4. Quantification of Infection Intensity

The methodology was used according to Pohl [24], briefly, spores in samples from each hive were counted in five fields of view. Based on their average value, the bee colony was classified into one of 4 categories of bee infestation according to the key: 0 spores/slide—negative sample; 1–19 spores/slide—weak *Nosema* infection (+); 20–100 spores/slide—moderate *Nosema* infection (++); above 100 spores/slide—strong *Nosema* infection (+++). The presence of infection in the apiary was calculated as the percentage of infected colonies in relation to the total number of examined colonies.

### 2.5. DNA Extraction

Frozen samples of the liquid fraction of the honeybee abdomen homogenate were thawed at room temperature prior to analysis.

DNA isolation was performed using the DNA-sorb-AM kit (AmpliSens^®^, Moscow, Russia), designed for the extraction and purification of nucleic acids from tissues, according to the manufacturer’s instructions. The sample was added to microtubes containing glass (0.5 mm) and zirconium beads (1.0 mm) ensuring better mechanical disruption of spores. After being covered with lysis solution, they were homogenized for 2 × 45 s at 6500 rpm using a Precellys 24 device (Berlin Technologies, GmbH, Berlin, Germany). If the extracted samples were not processed immediately, they were stored in a freezer at −20 °C until the next analysis.

### 2.6. PCR Amplification

For the detection of *Nosema*, we used duplex PCR, where the mix consisted of PCR water, FirePol^®^ Master Mix (Solis BioDyne, Tartu, Estonia, Cat. No. 04-11-00125), and 10 µM of primers [25] specific for both *Nosema* species (Table 1). Amplicon sizes obtained with the primer pairs were 321 bp for the *N. apis*-specific primers (APIS FOR/REV) and 218 bp for the *N. ceranae*-specific primers (MITOC FOR/REV), as described by Martín-Hernández et al. [25]. The template DNA was then added to the mix and thoroughly homogenized using a vortex.

Although multiplex (duplex) PCR assays may require a higher amount of template DNA to ensure positive amplification, we followed the validated protocol of Malčeková et al. [26], which reports adequate sensitivity for detecting both *Nosema* species. The precise limit of detection was not experimentally evaluated in our laboratory; however, the assay consistently amplified positive controls and showed full concordance with microscopic examination in samples with moderate and high spore loads. This supports the robustness of the method for the purposes of epidemiological screening.

Duplex PCR was performed using a VWR RISTRETTO thermocycler. Initial denaturation was 4 min at 95 °C, followed by 28 cycles of denaturation at 95 °C (25 s), annealing at 58 °C (45 s) and polymerization at 72 °C (2 min). The final extension step was 7 min at 72 °C. To prove the presence of DNA in the examined samples, we used gel electrophoresis, using a 1.5% agarose gel. After evaluating the results using a UV transilluminator, the DNA concentration was measured using a NanoDrop and the PCR products were sent for sequencing. The obtained sequences were compared with the sequences deposited in the gene bank using the BLAST program https://blast.ncbi.nlm.nih.gov/Blast.cgi (accessed on 21 April 2025).

### 2.7. Statistical Analysis

For the purposes of modelling the correlation between bee population density and colony infestation rate, only sample results from beekeepers who had 30 or more colonies at one site were included in the analysis. This inclusion threshold was set to ensure sufficient within-apiary replication for estimating the relative proportion of positive colonies, to reduce stochastic variability associated with small hobby apiaries, and to obtain a more stable epidemiological signal at each location. Larger operations also tend to follow more consistent management practices, which improves comparability across districts and strengthens the interpretability of density-dependent effects. This corresponded to the number of examined samples (colonies) of 1114 in 2022, 980 in 2023, and 991 in 2024. This filtering was employed to ensure a more objective comparison of districts based on the size of the apiaries as well as the minimum number of samples tested. Since the data were collected during the years 2022 to 2024, we calculated the average beekeeping density for districts over this period. The data originated from the Central Register of Beekeeping Colonies.

Differences in the number of positive samples between districts and years were assessed using analysis of variance and the Kruskal–Wallis test. Differences in the distribution of the relative proportion of positive samples between years were tested using the Kruskal–Wallis test.

The relationship between the relative proportion of positive colonies and beekeeping density was analysed using Generalized Estimating Equations (GEE) with a Gamma distribution and a logarithmic link function. The model was fitted using 21 clusters (districts) with a maximum cluster size of 9. The district was specified as a cluster with an autoregressive correlation structure (AR1). Linear and quadratic effects of colony density and the year factor were included in the model, with the significance of the predictors assessed by the Wald χ^2^ test. Analyses were performed in R environment (version 4.4.3) [27] using the “geepack” package [28].

The relationship between bee colony density (colonies per km^2^) and the proportion of positive samples was analysed using piecewise (segmented) linear regression. First, a simple linear regression model was fitted with the relative proportion of positive samples as the dependent variable and bee colony density as the independent variable.

Subsequently, the model was extended using the segmented package in R to estimate a potential breakpoint in bee colony density. One breakpoint (npsi = 1) was specified, allowing the slope of the regression line to differ before and after the estimated threshold value.

The model provided an estimate of the breakpoint (ψ) as well as the regression slopes on both sides of this point. The significance of the change in slope was evaluated using Wald-type tests implemented within the segmented framework.

Predicted values from the segmented model were used to visualise the fitted piecewise regression line together with the observed data [29].

Graphical outputs were generated using the “ggplot2” [30] and “ggthemes” [31] packages in R environment.

## 3. Results

### 3.1. Microscopic and Molecular Analysis of Nosema spp.

A total of 6221 samples taken from 43 districts in Slovakia were microscopically examined between 2022 and 2024. Of these, 4621 (74.3%) samples were positive, and 1600 (25.7%) samples were negative for the presence of *Nosema*.

**Figure 1:**
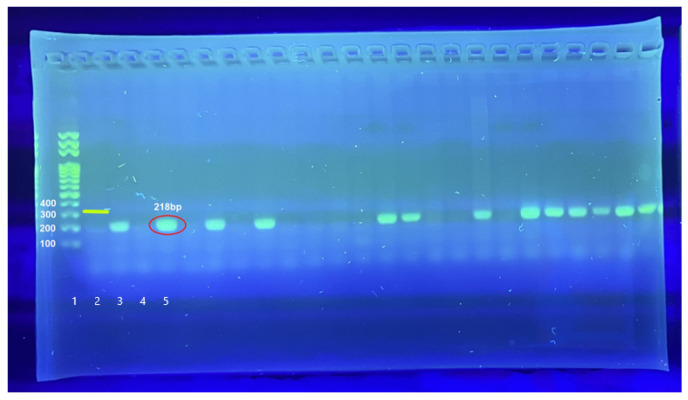
Visualisation of positive samples of *N. ceranae* using gel electrophoreses and UV transilluminator. Lane 1: molecular weight marker (100 bp ladder). Lane 2: illustration of positive control for *N. apis* (321 bp). Lane 3: positive control for *N. ceranae* (218 bp). Lane 4: negative control (no-template control) showing no amplification. Lanes 5–25: representative positive samples showing amplification of the *N. ceranae*-specific amplicon (218 bp) and negative samples.

Microscopic examination was confirmed by PCR analysis of all samples. All samples that underwent DNA isolation, followed by PCR amplification, were analysed using agarose gel electrophoresis and visualized with a UV transilluminator. Of the 6221 samples tested, 4621 (74.3%) were positive for *N. ceranae* (95% CI 73.18–75.35%); 1600 (25.7%) were negative. No samples were positive for *N. apis*—the upper 95% limit for the prevalence of *N. apis* is 0.048% (Clopper–Pearson). Figure 1 shows an example of the presence of *N. ceranae* by visualization on agarose gel.

### 3.2. Geographical Spread of N. ceranae Infection

**Figure 2:**
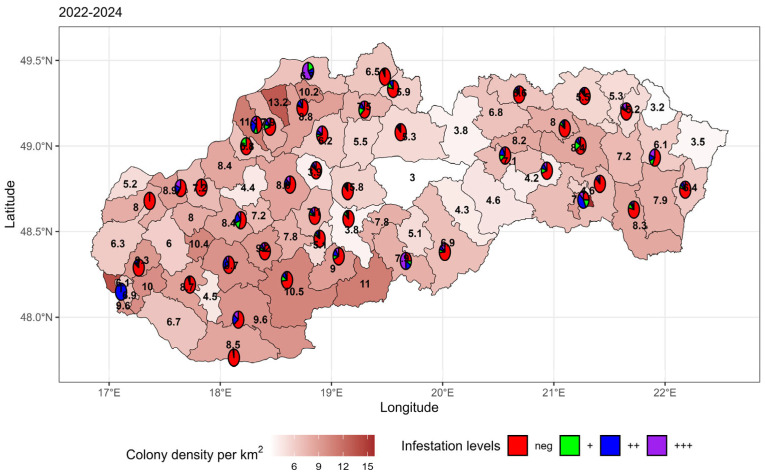
Comparison of *N. ceranae* infestation in honeybee colonies and beekeeping density in Slovakia (2022–2024).

To express geographical spread, all analysed samples (n = 6221) were included in the statistics, collected from 43 districts of Slovakia, of which 4621 samples were positive for the presence of *N. ceranae,* and 1600 samples were negative. Overall comparison shows that high-density districts (>10 colonies/km^2^) exhibit consistent increases in positivity severity (++/+++), indicating a strong correlation between density and infestation severity (Figure 2). High-density regions (e.g., central and southern districts) are transitioning from primarily weak (green/+) positivity in 2022 to strong (purple/+++) positivity by 2024. Progressive spread of weak positivity (green/+) into lower-density regions shows that even areas with intermediate densities are becoming more affected over time.

The prevalence of pathogens is consistently higher in northwestern central Slovakia and southern Slovakia, where the density is highest. However, the eastern and northeastern regions, initially dominated by negative samples (red), show an emerging spread of positivity as time progresses.

### 3.3. N. ceranae Infestation Levels Across Slovak Districts

**Figure 3:**
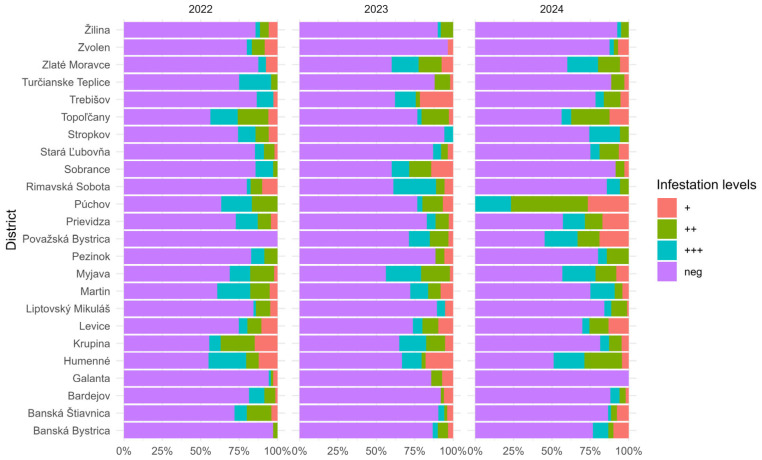
Percentage distribution of *N. ceranae* infestation levels across Slovak districts (2022–2024). The stacked bar chart illustrates the percentage distribution of infestation levels (0 spores/slide—negative sample; 1–19 spores/slide—weak *Nosema* infection (+); 20–100 spores/slide—moderate *Nosema* infection (++); above 100 spores/slide—strong *Nosema* infection (+++) in honeybee colonies across Slovak districts from 2022 to 2024. Infestation severity shows a progressive increase over time, with more districts reporting moderate (++) and severe (+++) positivity in 2024.

A total of 24 districts (Table S1) were evaluated in this section, with the condition that samples were collected in each district every year (2022, 2023, and 2024). In 2022, 1466 samples were examined, of which 1113 were positive and 353 were negative. In 2023, 1364 samples were examined, of which 1072 were positive and 292 were negative. In 2024, 1488 samples were examined, of which 1101 were positive and 387 were negative. The proportion of samples positive for *N. ceranae* varied between districts and years (Figure 3).

The results of the linear model did not show statistically significant differences between districts (F_(23,216)_ = 1.25, *p* = 0.21), between individual years (F_(2,216)_ = 0.08, *p* = 0.92) or their interaction (F_(46,216)_ = 0.15, *p* = 1.00).

Similarly, the Kruskal–Wallis test did not show significant differences between districts within individual years: in 2022 (χ^2^ = 21, df = 23, *p* = 0.60); in 2023 (χ^2^ = 18, df = 23, *p* = 0.80); nor in 2024 (χ^2^ = 22, df = 23, *p* = 0.50). These results indicate that no statistically significant differences in the number (or proportion) of positive samples were observed between districts during the monitored period.

### 3.4. N. ceranae Infestation Levels for Samples Collected from Areas with over 30 Beehives

**Figure 4:**
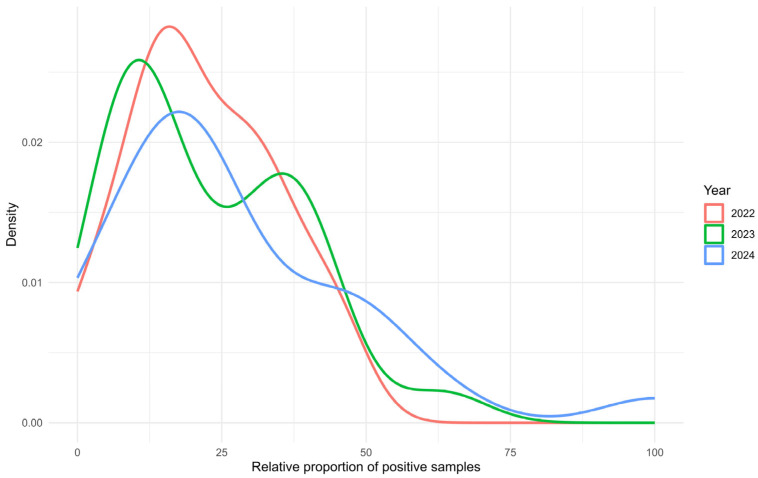
Density distribution of positive sample proportions over time (2022–2024). Red (2022): Distribution of positive sample proportions in 2022, peaking at low positivity rates (<25%), green (2023): Distribution for 2023, showing a shift towards higher positivity rates (25–50%), blue (2024): Distribution for 2024, with positivity rates more spread out and peaking in intermediate to high positivity ranges (45–70%). The *x*-axis represents the relative proportion of positive samples (in percentage), and the *y*-axis shows the density distribution, indicating where many positive sample proportions are concentrated. Each curve represents the data for a specific year.

From analyses of samples from apiaries with more than 30 colonies, we expressed the percentage of positive samples to negative samples for individual districts (Figure 4). These statistics include data from locations where we examined more than 30 samples at one location. In 2022, the data included represented 1114 samples, of which 247 were positive and 867 were negative. In 2023, the data included represented 980 samples, of which 219 were positive and 761 were negative. In 2024, the data included represented 991 samples, of which 263 were positive and 728 were negative.

There was no statistically significant difference between the groups (Kruskal–Wallis χ^2^ = 0.6, df = 2, *p* = 0.70). Significant year-to-year differences in districts were therefore not confirmed, but based on the graphical representation, certain trends can be observed.

In 2022, the density curve peaked at lower levels of apiary farm positivity (10–20%). Most districts recorded relatively few *N. ceranae*—positive samples, with a sharp decline in density beyond 25%. This pattern indicates that the pathogen’s spread was relatively limited during this year.

In 2023, the peak density shifted to a higher positivity range (25–35%). The broader distribution reflects an overall increase in positivity compared with 2022. The curve flattened slightly above 50%, suggesting that moderate positivity became more common, whereas extreme values (>75%) remained rare.

In 2024, the density curve broadened substantially, indicating increased variability in positivity levels across districts. The distribution flattened further, with overall proportions of positive samples higher than in previous years. A secondary rise near 75–100% suggests that some districts experienced very high proportions of infected colonies per apiary. The rightward shift from 2022 to 2024 reflects a progressive increase in *N. ceranae* positivity, while the broadening of the curves highlights growing heterogeneity among districts. By 2024, positivity levels above 75% were observed in some districts, underscoring the potential risk of severe disease outbreaks in specific regions.

### 3.5. Model of Positivity Development

**Figure 5:**
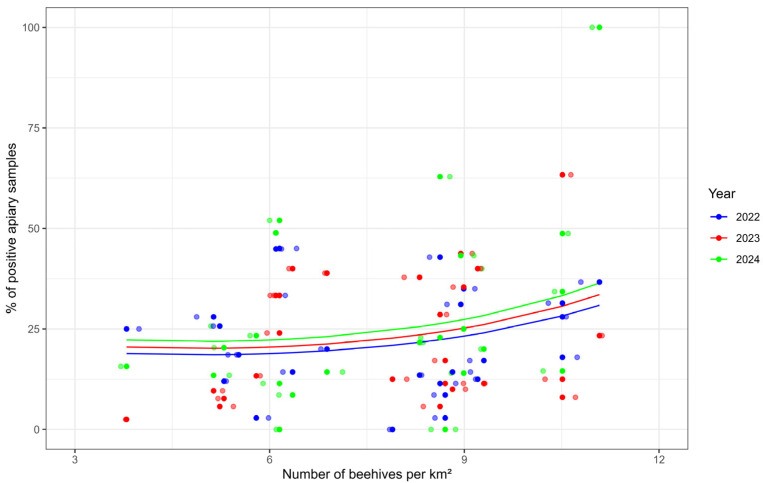
Relationship between positivity of colonies and beekeeping density in the region.

To evaluate the relationship between the relative proportion of positive colonies on *Nosema* and beekeeping density, a generalized linear model analysis with clustering by district (GEE—Generalized Estimating Equations) with Gamma distribution and logarithmic link function was used (Figure 5).

These statistics include data from locations where we examined more than 30 samples at one location. In 2022, the data included represented 1114 samples, of which 247 were positive and 867 were negative. In 2023, the data included represented 980 samples, of which 219 were positive and 761 were negative. In 2024, the data included represented 991 samples, of which 263 were positive and 728 were negative.

The model included both linear and quadratic terms for colony density and the year factor. The correlation structure between district replicates was modelled as autoregressive (AR1).

The full model showed that the linear effect of colony density was statistically significant (Waldo χ^2^ = 4.35, *p* = 0.037), while the quadratic term (χ^2^ = 0.37, *p* = 0.54) and year (χ^2^ = 1.37, *p* = 0.24) were not significant. After removing these insignificant variables (terms), the model remained with the beekeeping density variable alone, which retained statistical significance (Waldo χ^2^ = 4.35, *p* = 0.037).

According to the estimate of the parameter for beekeeping density (β = 0.0807, SE = 0.0387, 95% CI [0.005, 0.157], *p* = 0.037), we can interpret that when the beekeeping density increases by one colony per km^2^, the relative proportion of positive bee colonies increases by approximately 8.4%.

This interpretation is based on the log-link function used in the model: exp(β) − 1 = exp (0.0807) − 1 ≈ 0.084, i.e., an 8.4% increase in the expected value of the dependent variable.

The quadratic term was not statistically significant, but visual inspection of the residuals and prediction curves suggests a slight curvature of the dependence.

The segmented regression identified a breakpoint at 10.41 colonies/km^2^ (SE = 0.19; 95% CI: 10.03–10.79). Below this threshold, the slope was 0.74 (SE = 1.23; 95% CI: −1.72 to 3.20; *p* = 0.552), indicating no significant association. Above the breakpoint, the slope increased to 43.07 (SE = 19.25; 95% CI: 4.71–81.44), demonstrating a statistically significant increase in the proportion of positive samples at higher colony densities.

Based on this, our recommendation to the beekeeping community is to avoid exceeding this density when placing colonies in the landscape. For the period between 2022 and 2024, the relationship between positivity for *Nosema* and beekeeping density in the Slovak Republic can be described by the equation:

E[Y] = exp (2.53899 + 0.08069 × HV) − 0.1

where E[Y] represents the expected positivity rate in percent, and HV represents the colony density in colonies per km^2^.

This study provides novel insights by demonstrating the utility of hive bottom debris as a diagnostic resource for *Nosema* detection, particularly in high-density apiaries. This approach offers a cost-effective and non-invasive alternative for routine monitoring.

## 4. Discussion

This nationwide study demonstrates that winter bottom-hive cadavers represent a reliable and informative matrix for assessing the intensity of *Nosema ceranae* infestation at the onset of the beekeeping season. The results confirm the near-exclusive presence of *N. ceranae* in Slovak honeybee colonies and refine the current understanding of how beekeeping density relates to infection pressure. Although the statistical relationship between colony density and infestation was significant, the effect size was modest, indicating that density contributes to the risk of infection but is not the principal driver of its spatial variability. These findings position density as an amplifying factor rather than a sole determinant of pathogen prevalence. Such patterns are consistent with broader European trends, where *N. ceranae* has become the dominant microsporidian across *Apis mellifera* populations, accompanied by marked spatial heterogeneity [32,33,34].

The diagnostic value of winter cadavers lies in their ability to capture the cumulative infection load from the previous season. Because colonies in January and February contain long-lived winter bees and are largely broodless, the confounding influence of age structure is minimized, allowing winter cadavers to reflect genuine pathogen pressure rather than short-term fluctuations in the forager population. This makes the method particularly relevant in systems where decisions about colony selection for queen rearing must be made prior to the active season. Winter sampling therefore provides veterinarians and breeders with timely, actionable information that cannot be obtained from sampling conducted later in the season. Moreover, complementing microscopy with molecular diagnostics greatly improves species-level resolution and is essential for distinguishing *N. ceranae* from *N. apis* [35].

The observed association between beekeeping density and *N. ceranae* positivity supports the idea that colony aggregation facilitates opportunities for pathogen transmission through overlapping foraging ranges and shared environmental resources. However, the substantial variation among districts with similar densities suggests that environmental and management factors strongly modulate the measurable effect of density. Climatic conditions, particularly winter temperature patterns and the onset of spring, may influence the survival and proliferation of *Nosema* spores both within and outside the colony. Likewise, nutritional conditions shaped by landscape structure—such as the dominance of monocultures, the availability of semi-natural habitats, and temporal gaps in forage availability—may significantly affect colony resilience to infection. Additional factors such as migratory beekeeping practices, Varroa destructor infestations, associated viral loads, and exposure to agricultural pesticides likely contribute further to regional variation in infestation intensity. The synergistic impact of Varroa-associated viruses on overwintering colony survival has been demonstrated in several European regions [36], reinforcing the notion that *N. ceranae* often interacts with other stressors rather than acting alone. Furthermore, natural infection studies have shown that *N. ceranae* may induce physiological exhaustion and contribute to colony collapse in specific ecological contexts [37].

Given these complexities, future epidemiological analyses would benefit from integrating climatic datasets with standardized forage indicators and consistent health metrics such as Varroa and virus levels. Statistical modelling approaches that allow for hierarchical or non-linear relationships, including mixed-effects models or generalized additive models, would be well suited to disentangle the relative contributions of density, environmental context, and management practices. Incorporating these variables will enable a more mechanistic understanding of nosemosis dynamics and improve the predictive value of landscape-level surveillance. Future comparisons should also be interpreted within the broader European epidemiological framework, where both climatic and genetic factors influence *N. ceranae* prevalence and infection dynamics [32,33,34].

From a management perspective, the findings underscore the importance of maintaining winter cadaver screening as a key pre-season diagnostic tool. In regions exhibiting elevated positivity or where colony densities approach the higher end of the observed range, complementary early-spring screening of live bees may further improve the detection of emerging infection increases. Where practical constraints make it difficult to avoid higher concentrations of colonies, implementing targeted nutritional support and rigorous Varroa management may help offset the increased risk. For queen-rearing programs, a combined interpretation of PCR positivity and microscopically determined infestation intensity appears to be the most robust approach for selecting colonies with stable, low infection pressure. Ensuring that PCR assays use validated species-specific primer sets is essential for diagnostic reliability across laboratories [35].

The strengths of this study include its large sample size, uniform timing of sample collection, and dual-method confirmation of *Nosema* detection through microscopy and duplex PCR. Nonetheless, some limitations must be acknowledged. The limit of detection for the multiplex PCR assay was not experimentally quantified in our laboratory, although the method consistently amplified positive controls and aligned with microscopic results in samples with moderate and high spore loads. In addition, the absence of high-resolution environmental data limits the ability to fully attribute spatial differences in infestation to specific ecological or management factors. The incomplete annual representation of smaller hobby apiaries may also introduce mild sampling bias, although the scale of the study partly mitigates this concern. 

Future research should focus on multi-year, colony-level panels that integrate winter cadaver data with early-season sampling of live bees and standardized Varroa and virus measurements. Coupling epidemiological data with high-resolution environmental and land-use indicators will allow a deeper exploration of causal pathways underlying *N. ceranae* spread and persistence. Experimental studies, such as targeted density-reduction trials or forage-improvement interventions, would add mechanistic evidence to support observational findings. Furthermore, laboratory comparisons of singleplex and duplex PCR across dilution series would allow precise quantification of detection limits, strengthening diagnostic interpretation in low-intensity infestations. 

In conclusion, winter bottom-hive cadavers offer a powerful pre-season window into colony health and provide valuable insights for management decisions related to disease control and queen breeding. Although colony density has a measurable effect on infestation levels, the results clearly show that environmental, nutritional, and management factors exert substantial influence on the epidemiology of *Nosema ceranae*. This aligns with extensive evidence identifying *N. ceranae* as a major pathogen in European honeybee populations, especially when acting synergistically with pesticides, Varroa mites, and viral infections [32,37,38,39]. A more integrated approach—combining improved diagnostic standardization, enhanced environmental data, and targeted health interventions—will be essential for strengthening honeybee resilience and optimizing apicultural decision-making.

## 5. Conclusions

Winter bottom beehive debris represents a practical and reliable material for assessing the intensity of *Nosema ceranae* infestation in honeybee colonies. Although increased beekeeping density may contribute to higher infestation pressure, its effect, although statistically significant, is insufficient to fully explain regional differences in infestation intensity. Effective management of nosemosis therefore requires an integrated approach that considers environmental conditions, beekeeping practices, and routine monitoring of infestation levels using winter debris samples.

## Figures and Tables

**Table 1:** Composition and amount of PCR mix ingredients used for PCR amplification of *Nosema* samples.

PCR Mix Composition	Volume
PCR water	11.5 µL
Firepol Master Mix (Solis Biodine)	4 µL
APIS FOR (5′-GGGGCCATGTGTTTGACGTACTATGTA-3′)	0.5 µL
APIS REV (5′-GGGGGGCGTTTAAAAATGTGAAACAACTATG-3′)	0.5 µL
MITOC FOR (5′CGGCGACGATGATGATGATGAAAATATTAA-3′)	0.5 µL
MITOC REV (5′-CCCGGTCATTCTCAAAAAAAACCG-3’)	0.5 µL
Template	2.5 µL

## Data Availability

The original contributions presented in this study are included in the article/Supplementary Material. Further inquiries can be directed to the corresponding author.

## Supplementary Materials

The following supporting information can be downloaded at: https://www.mdpi.com/article/10.3390/microorganisms14030694/s1, Table S1: Percentage of samples by infestation intensity, districts, and years; Figure S1: Beekeeping in Slovakia in 2022–2024.
