# Supplementary material for: Winter Bottom Beehive Cadavers as a Tool for Assessing Nosema ceranae Infestation Intensity in Honeybee Colonies in Regions with Different Beekeeping Densities in Slovakia
Source: Microorganisms. 2026 Mar 19;14(3):694. doi: 10.3390/microorganisms14030694 (PMC13029231; doi:10.3390/microorganisms14030694)
Supplement: Supplementary file 1 [file microorganisms-14-00694-s001.zip › microorganisms-4137852-Supplementary Figure S1.pdf]

Winter Bottom Beehive Cadavers as a Tool for Assessing *Nosema ceranae* Infestation Intensity in Honeybee Colonies in Regions with Different Beekeeping Densities in Slovakia

Simona Hriciková [1], Martin Staroň [2], Lucia Sabová [3], and Monika Sučik [1*]

Supplementary material

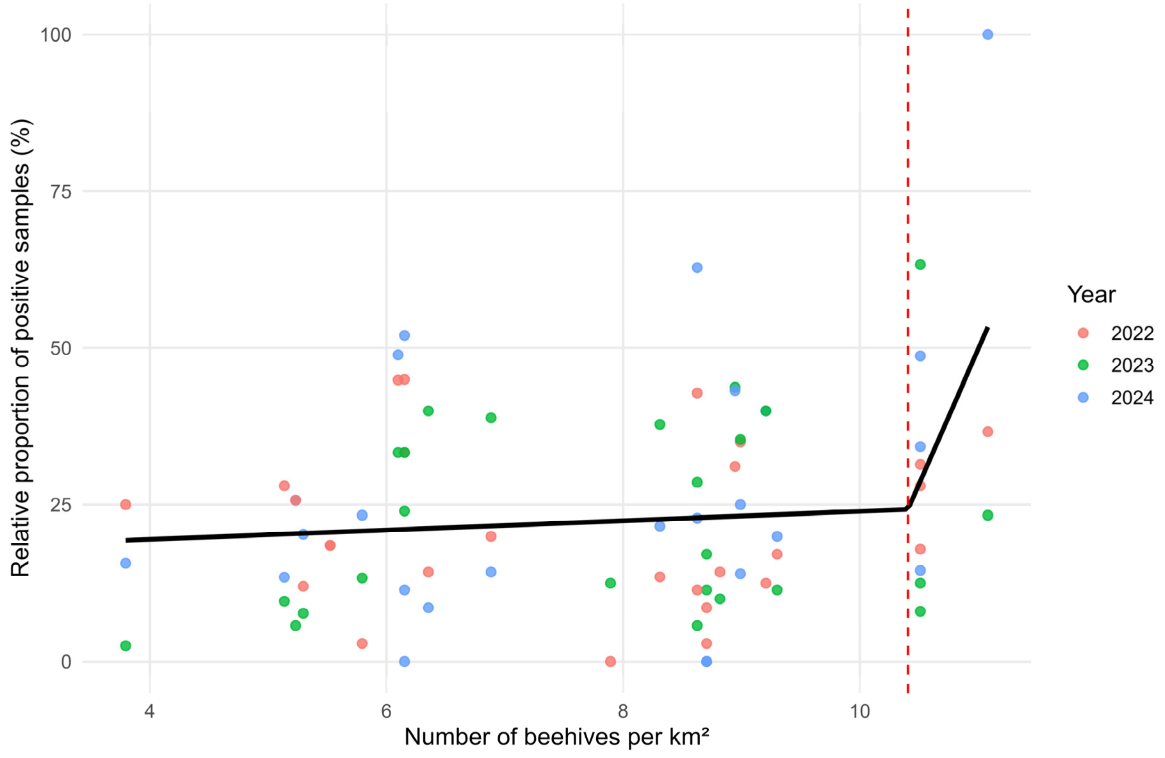

Figure S1. Beekeeping in Slovakia in 2022–2024.
